# Student Entrepreneur Behavior (SEB) : A Bibliometric Analysis

**DOI:** 10.12688/f1000research.175425.1

**Published:** 2026-05-25

**Authors:** Munawir Nasir Hamzah, Lim Sanny, Diena Dwidienawati, Agustinus Bandur

**Affiliations:** 1 Doctor of Research in Management, BINUS University, West Jakarta, Jakarta, 11530, Indonesia

**Keywords:** Student entrepreneurial behavior; Entrepreneurship education; Bibliometric analysis; Entrepreneurial intention; Theory of Planned Behavior

## Abstract

**Background:**

Student Entrepreneurial Behavior (SEB) has gained increasing attention within entrepreneurship education research. However, existing studies remain fragmented and predominantly emphasize entrepreneurial intention rather than observable behavior.

**Objective:**

This study aims to provide an exploratory bibliometric mapping of Student Entrepreneurial Behavior (SEB) research, identifying its intellectual structure, thematic evolution, and research gaps within higher education contexts.

**Methods:**

A bibliometric analysis was conducted on 31 Scopus-indexed publications published between 2020 and 2024. Data were retrieved using Publish or Perish and analyzed using VOSviewer, employing performance analysis and keyword co-occurrence mapping techniques.

**Results:**

The findings indicate that Student Entrepreneurial Behavior (SEB) research is still in an early developmental stage, characterized by fluctuating publication trends, geographic concentration, and a predominance of cross-sectional designs. Thematic clustering reveals several key research streams, including individual determinants, entrepreneurship education outcomes, social influences, and measurement development.

**Conclusions:**

This study highlights the persistence of the intention-behavior gap and underscores the need for longitudinal, mixed-method approaches and more robust operationalization of entrepreneurial behavior as a measurable educational outcome. The findings provide initial insights to guide future research and curriculum development in entrepreneurship education.

## Introduction

The contemporary global economy is increasingly shaped by rapid technological disruption, shifting labor markets, and the growing demand for innovation-driven growth. These transformations have significant implications for higher education, particularly in aligning curriculum design with evidence-based policy and institutional decision-making. Within this context, entrepreneurship has been widely recognized as a critical driver of economic development, job creation, and broader societal transformation ( Wang et al., 2024). Accordingly, universities are no longer viewed merely as sites of knowledge transmission, but as entrepreneurial ecosystems that cultivate students’ mindsets, competencies, and behaviors. This shift reflects a transition in entrepreneurship education from a predominantly cognitive orientation toward a more practice-oriented approach, emphasizing observable entrepreneurial behaviors as key learning outcomes.

In higher education, entrepreneurship education is increasingly framed within an outcome-based learning paradigm, which aligns with contemporary educational quality assurance and accreditation standards. Educational success is thus assessed not only through students’ entrepreneurial knowledge or intentions, but through their demonstrated capacity to engage in entrepreneurial activities during their academic trajectories. Policymakers and educators globally acknowledge that fostering Student Entrepreneurial Behavior (SEB) is essential, as it represents the translation of entrepreneurial learning into practice and bridges the long-standing divide between classroom instruction and real-world entrepreneurial engagement. Evidence from the Global Entrepreneurship Monitor (GEM) 2023 report further highlights the relevance of this issue, indicating that youth entrepreneurship (ages 18–34) constitutes a substantial proportion of new business creation worldwide, with university students emerging as a particularly promising group due to their access to institutional resources, networks, and structured learning environments ( Global Entrepreneurship Monitor, 2023). Despite sustained investments in entrepreneurship education programs, incubators, and accelerator initiatives, a persistent gap remains between students’ entrepreneurial intentions and their actual entrepreneurial behavior ( Al-Qadasi et al., 2024; Neneh & Dzomonda, 2024), indicating unresolved challenges in instructional design and learning transfer within higher education contexts.

Student Entrepreneurial Behavior (SEB) refers to the observable actions, decisions, and patterns of engagement through which students pursue entrepreneurial opportunities during their university education, and can be conceptualized as a measurable learning outcome within entrepreneurship curricula. Importantly, Student Entrepreneurial Behavior (SEB) is conceptually distinct from entrepreneurial intention, which reflects a cognitive commitment to future entrepreneurial activity rather than enacted behavior. Student Entrepreneurial Behavior (SEB) encompasses concrete forms of entrepreneurial participation, including opportunity identification, resource mobilization, venture creation, and business implementation ( Manzi-Puertas et al., 2024). Positioning Student Entrepreneurial Behavior (SEB) as a core learning outcome of entrepreneurship education enables a more precise assessment of educational effectiveness, shifting evaluative focus from attitudinal change toward measurable behavioral enactment and learning transfer, thus strengthening constructive alignment between learning objectives, instructional strategies, and assessment practices in higher education.

From an educational perspective, understanding the factors that facilitate or constrain the transition from entrepreneurial intention to actual behavior is critical for the design of effective pedagogical strategies, curriculum frameworks, and experiential learning models. Student Entrepreneurial Behavior (SEB) serves as an integrative indicator of learning transfer, reflecting the extent to which entrepreneurship education translates pedagogical inputs into practical entrepreneurial action. Engagement in entrepreneurial activities during university education supports the development of adaptive thinking, resilience, innovation capacity, and strategic decision-making competencies that remain valuable across diverse career pathways ( Boubker, 2024), thereby contributing to graduate employability and lifelong learning competencies. Moreover, student-initiated ventures contribute to economic growth, employment creation, and technological advancement, underscoring the broader societal relevance of Student Entrepreneurial Behavior (SEB) beyond individual educational achievement. Insight into Student Entrepreneurial Behavior (SEB) trends therefore holds significant potential to inform curriculum design, institutional support mechanisms, and policy interventions aimed at enhancing the impact of entrepreneurship education ( Diepolder et al., 2025) particularly in aligning higher education outcomes with national education development agendas.

Although research on student entrepreneurship has expanded substantially over the past two decades, much of the existing literature remains concentrated on entrepreneurial intention, psychological antecedents, institutional support mechanisms, and intention-based theoretical frameworks, most notably the Theory of Planned Behavior (TPB). Extensions of Theory of Planned Behavior (TPB) have incorporated constructs such as self-efficacy, passion, opportunity recognition, and social capital ( Shen et al., 2025; Liu et al., 2025). However, several limitations persist. First, despite the dominance of intention-based models, relatively few studies explicitly conceptualize and examine Student Entrepreneurial Behavior (SEB) as an educational outcome in its own right, particularly through behavior-based indicators, thereby limiting its integration into educational evaluation systems. Second, the prevalence of cross-sectional research designs constrains understanding of the temporal development of entrepreneurial behavior, which is essential for longitudinal assessment of student learning progression. Third, the literature exhibits a strong geographic concentration, with limited representation from non-Western and underrepresented higher education contexts, thereby reducing the generalizability and contextual relevance of existing findings.

Methodological challenges further complicate knowledge accumulation in this field. Concerns regarding measurement validity and reliability remain salient, as many instruments lack rigorous validation, thereby limiting comparability across studies ( Wang and Sahid, 2024a), and weakening the robustness of educational outcome assessment. Collectively, these issues point to the absence of a consolidated research agenda that integrates educational, behavioral, and contextual perspectives on Student Entrepreneurial Behavior (SEB). While individual-level determinants have received considerable attention, meso-level influences related to institutional ecosystems and macro-level factors such as policy environments and cultural norms remain underexplored, highlighting the need for a more systematic and integrative mapping of the field.

Given the fragmented and interdisciplinary nature of Student Entrepreneurial Behavior (SEB) research, there is a clear need for systematic synthesis to map the intellectual structure and developmental trajectory of this emerging field, particularly to inform evidence-based educational reform and curriculum innovation. Bibliometric analysis provides a robust and transparent approach for addressing this need by enabling quantitative examination of publication trends, citation patterns, collaborative networks, and thematic structures. Unlike narrative reviews, which may be shaped by subjective selection and interpretation, bibliometric methods offer a data-driven overview of how Student Entrepreneurial Behavior (SEB) research has evolved, which themes dominate scholarly attention, and where critical gaps persist, thereby supporting more transparent and reproducible research synthesis in line with open science principles.

While prior bibliometric studies have examined entrepreneurship education more broadly ( Gabbianelli et al., 2021), no comprehensive bibliometric analysis has explicitly framed Student Entrepreneurial Behavior (SEB) as a distinct learning outcome within entrepreneurship education. Therefore, this study aims to provide a systematic bibliometric mapping of Student Entrepreneurial Behavior (SEB) research published between 2020 and 2024. Specifically, it identifies key publication trends, influential authors and sources, collaborative networks, and thematic developments within the field. By doing so, the study captures recent shifts in research priorities following global disruptions such as the COVID-19 pandemic and contributes to advancing outcome-oriented perspectives in entrepreneurship education ( Jiatong et al., 2022), with explicit implications for evidence-based curriculum development, teaching innovation, and higher education quality enhancement.

## Literature Review

The concept of entrepreneurship has evolved significantly, transitioning from a purely economic activity to a multifaceted phenomenon involving innovation, leadership, and social impact. At its core, entrepreneurship is understood as the capacity of individuals to identify, create, and exploit business opportunities while managing associated risks. This broader conceptualization emphasizes the role of entrepreneurs not only as business initiators but also as change agents who drive innovation and sustainability within economies ( Murad et al., 2024b; Wang et al., 2024).

Within this broader domain, **Entrepreneurial Intention (EI)** has emerged as a pivotal construct, widely explored through the lens of Ajzen’s Theory of Planned Behavior (TPB). This theory posits that entrepreneurial intentions are shaped by attitudes toward entrepreneurship, subjective norms, and perceived behavioral control ( Razi-ur-Rahim et al., 2024; Liu et al., 2025). While EI provides a theoretical framework to understand motivations, the actualization of entrepreneurial behavior often diverges from intentions, revealing a behavioral gap that requires further exploration ( Relente & Capistrano, 2025).

A growing subset of research focuses on **Student Rntrepreneurial Behavior (SEB)**, highlighting students as an emerging demographic within the entrepreneurial landscape. Student Entrepreneurial Behavior (SEB) encompasses attitudes, decisions, and actions taken by students toward starting and managing entrepreneurial ventures, often influenced by internal motivations, educational background, and external support systems ( Boubker, 2024; Ahmed et al., 2025). Multiple studies indicate that while students demonstrate strong entrepreneurial intentions, these rarely translate into concrete actions due to barriers such as limited access to funding, lack of mentorship, and institutional constraints ( Diepolder et al., 2025; Manzi-Puertas et al., 2024).

In parallel, the role of **entrepreneurship education** has been extensively studied as a key enabler of Student Entrepreneurial Behavior (SEB). Findings suggest that well-designed curricula can enhance entrepreneurial mindset and skills, although the impact varies across institutional and cultural contexts ( Ayob, 2021; Bejinaru et al., 2023). However, few studies critically examine the long-term effects of such education or compare pedagogical approaches (e.g., experiential vs. theoretical learning). Furthermore, methodological inconsistencies and context-specific tools used to assess Student Entrepreneurial Behavior (SEB) have limited the comparability of findings across regions ( Soba et al., 2021; Wang and Sahid, 2024b).

Recent literature also emphasizes the need for **scale validation** and the development of reliable measurement instruments that can be applied cross-culturally. Many existing tools lack psychometric robustness or are validated only within narrow samples, typically limited to a single country or institution ( Alakaleek et al., 2023). Additionally, the predominance of cross-sectional studies limits understanding of the progression from intention to behavior, calling for longitudinal approaches ( Zhao, 2022; Jiatong et al., 2022).

Despite the growing body of knowledge, key gaps remain in the literature: (1) a lack of global synthesis of research trends and thematic focus; (2) limited attention to cross-cultural generalizability of findings; (3) underrepresentation of longitudinal and empirical studies linking intention to behavior; and (4) insufficient integration of institutional and policy-level factors that could support or hinder Student Entrepreneurial Behavior (SEB). These gaps underscore the need for a comprehensive bibliometric analysis to systematically review and synthesize global research on Student Entrepreneurial Behavior (SEB) and to inform future directions in this evolving field.

This study aims to identify global trends, thematic clusters, and methodological gaps in research on student entrepreneurial behavior during 2020-2024 through bibliometric analysis, in order to guide future research and policy formulation.

## Methodology

This study employed a bibliometric approach to provide an exploratory mapping of research on Student Entrepreneurial Behavior (SEB) published between 2020 and 2024. The analysis followed a transparent and replicable procedure adapted from established bibliometric protocols.

The Scopus database was selected as the primary data source due to its broad coverage of peer-reviewed international publications. Data were retrieved using Publish or Perish software by applying the following search query: TITLE (“student entrepreneurial behaviour” OR “student entrepreneurial behavior”). The search was restricted to journal articles and conference papers published in English between 2020 and 2024.

The initial results were screened based on relevance to Student Entrepreneurial Behavior (SEB) in higher education. Studies focusing solely on entrepreneurial intention without behavioral indicators were excluded. The final dataset consisted of 31 publications, reflecting the specificity of the search query and the emerging nature of the research domain.

Bibliographic data were exported in RIS format and analyzed using VOSviewer. The analysis included keyword co-occurrence mapping and thematic clustering, applying a full counting method with a minimum keyword occurrence threshold of two. The focus on title-based terms ensured conceptual precision, although it may limit the breadth of captured literature.

Given the relatively small dataset, this study adopts an exploratory bibliometric perspective, aiming to identify emerging patterns and research gaps rather than providing exhaustive generalization.

## Results

### Research Process and Data Identification

The research process in this study was conducted through six systematic stages aimed at identifying and analyzing global publications on Student Entrepreneurial Behavior (SEB) from 2020 to 2024. The initial stage involved subject limitation, where a general search using the keyword “Entrepreneurial Behaviour” was conducted in the Scopus database through the Publish or Perish application. Due to technical constraints allowing a maximum of 200 records per query, the searches were executed separately for each publication year. The total number of articles identified for each year was as follows: 2020 (190 articles), 2021 (197 articles), 2022 (200 articles), 2023 (200 articles), and 2024 (200 articles). These articles were filtered using citation-based relevance and H-index considerations to ensure academic quality.

The second stage involved a scope limitation, refining the search term to “Student Entrepreneurial Behaviour (SEB).” This search, which applied the same publication time frame and was also conducted using the Publish or Perish application, resulted in 1 article in 2020, 8 in 2021, 11 in 2022, 5 in 2023, and 6 in 2024, yielding a final sample of 31 eligible articles. The inclusion criteria limited the dataset to journal articles and conference papers, while excluding books and other non-peer-reviewed materials. Data collection was carried out from April 13 to April 21, 2025, and the selected bibliographic records were exported in RIS format to be processed further.

In the identification stage, the dataset was finalized for analysis. Descriptive statistics were applied to provide an overview of the publication distribution across years, citation frequency, country of origin, and the journals or publishers involved. These data were visualized using tables and graphs to enhance interpretation.

To map thematic trends and keyword relationships, the RIS data were analyzed using the VOSviewer software. The software was configured to create a map based on text data, with inputs sourced from reference manager files. The analysis focused on terms extracted from article titles, using the full counting method to measure frequency. A minimum occurrence threshold of two was set, allowing only keywords that appeared at least twice to be included in the mapping. Once configured, VOSviewer processed the data and generated co-occurrence maps that visually illustrated the clustering and interrelation of key research themes.

In the final stage, all 31 selected articles were reviewed in depth to identify existing research gaps and formulate suggestions for future investigation. This analysis revealed a number of underexplored areas, including limited use of longitudinal research designs, insufficient cross-cultural validation of Student Entrepreneurial Behavior (SEB) instruments, and a lack of empirical focus on the transition from entrepreneurial intention to action. The study concludes by emphasizing the need for future research to address these gaps and to promote stronger institutional, educational, and policy-level support for student entrepreneurship. The findings contribute to the advancement of the Student Entrepreneurial Behavior (SEB) research agenda and provide strategic direction for scholars and practitioners working in this field. Figure 1. Research workflow of the bibliometric analysis is referenced in this section.

**Figure 1.**
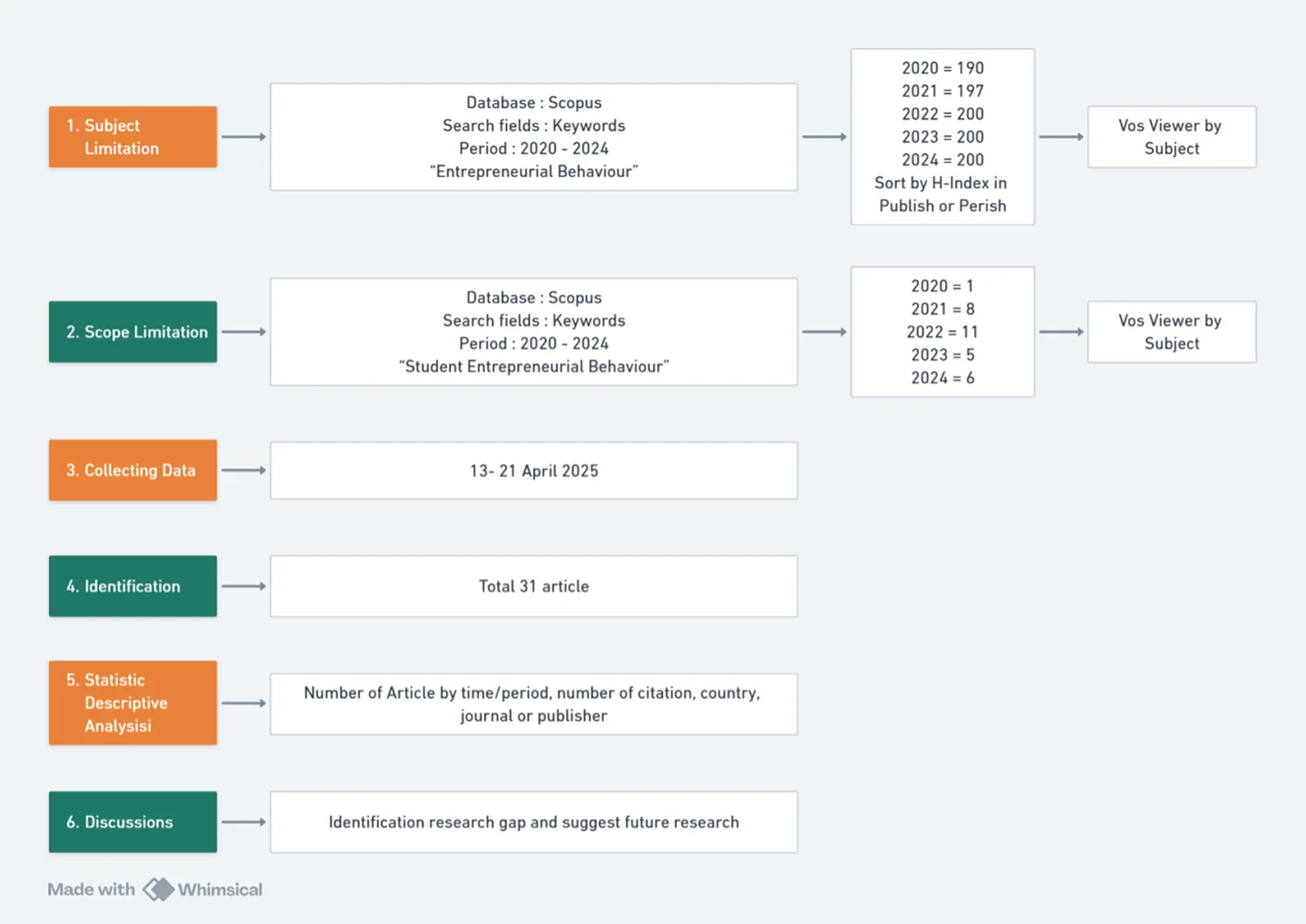
Research workflow of the bibliometric analysis. The figure illustrates the systematic process of data collection, screening, and analysis, including subject limitation, scope limitation, data collection period, identification of selected articles, descriptive statistical analysis, and research gap identification.

### Descriptive Statistic

Descriptive statistical analysis is conducted on the 31 articles that were obtained. Which is 28 articles come from journals, and the remaining 3 articles are from conference papers.

### Citation Count

The citation count for each article indicates how many times the article has been cited by other articles. In Table 1, 25 articles are displayed and sorted based on the highest citation count. The remaining 6 articles, which are not displayed, have a citation count of 0. The article titled “The impact of entrepreneurship education: A study of entrepreneurial outcomes” has the highest citation count, with a total of 35 citations.

**Table 1.** Citation Count.

Cites	Authors	Title	Year
35	W. Alakaleek	The impact of entrepreneurship education: A study of entrepreneurial outcomes	2023
25	A.H. Ayob	Entrepreneurship education, institutions and student entrepreneurship: a cross-country analysis	2021
21	M.L. Campos	Do university ecosystems impact student’s entrepreneurial behavior?	2021
19	M.A. Fauzi	The influence of transformational leadership on malaysian students’ entrepreneurial behaviour	2021
18	A. Majeed	Role of Entrepreneurial Passion between Entrepreneurial Attitude, Subjective Norms, Perceived Behavioral Control, and Entrepreneurial Intention: Measuring the Entrepreneurial Behavior of Pakistani Students	2021
13	P. Ataei	The process of adopting entrepreneurial behaviour: Evidence from agriculture students in Iran	2021
8	R. Bejinaru	Exploring the effectiveness of university agenda for developing students’ entrepreneurial behavior	2023
7	E. Nam	How does social media influence college students to recognize entrepreneurial opportunities? - Evidence from China	2021
6	M. Murad	Three stages of entrepreneurial university support and students’ entrepreneurial behavior: A statistical analysis using R Studio	2024
5	M. Fitouri	Does entrepreneurship education affect the choice between entrepreneurial and employee logic: a case study of Tunisian students	2023
5	A. Oktavio	Indonesian Students’ Entrepreneurial Behavior: The Effect of Creativity, Passion, Alertness And Intention	2023
4	W. Jiatong	From COVID-19 Pandemic to Entrepreneurial Behavior: The Mediating Effect of Proactive Personality and the Moderating Role of Anticipated Regret	2022
3	A.K.L.d. Rocha	Entrepreneurial behavior and education in times of adversity	2022
3	L. Zhao	On the Entrepreneurial Intention and Entrepreneurial Behavior Path of College Students Based on the Theory of Planned Behavior	2022
3	Y. Cheng	The Impact of Enterprise Management Elements on College Students’ Entrepreneurial Behavior by Complex Adaptive System Theory	2022
3	D. Yang	The Impact of Adaptive Learning in Entrepreneurial Behavior for College Students	2022
2	R.M. Barreto Colichi	Entrepreneurship and family support in nursing students from Brazil and Chile	2021
2	F. Wang	Validation of the Entrepreneurial Behaviour Scale among Vocational College Students in China, Using Exploratory Factor and Reliability Analysis	2024
1	J.A. Aguirre-González	University student’s entrepreneurial journey obstacles in costa rica	2020
1	M. Soba	Students’ entrepreneurial behaviour – An eight-construct scale validation: A scale adaptation study	2021
1	H. Zhang	Influence of Film Role on the Positive Development of College Students’ Entrepreneurial Behavior Using Positive Psychology	2022
1	S.H.N.A.b.M. Saupi	Entrepreneurial behaviours of final year students at vocational colleges toward interest in entrepreneurship	2022
1	Q.C. Bi	Proactivity, mindsets and the development of students’ entrepreneurial self-efficacy: behavioural skills as the catalyst	2022
1	J. Patel	Does my school teach me entrepreneurship? School entrepreneurship curriculum and students’ entrepreneurial intention: a serial mediation-moderation analysis	2024
1	D.D. Sedegah	Entrepreneurial intentions of tourism and hospitality students in Ghana: an application of the theory of planned behaviour	2024

### Country of the Publisher

Examining the country of origin of the publishers in the SLR articles helps us understand how geographic, social, economic, and policy factors in each country influence entrepreneurship research. This provides a broader perspective on student entrepreneurial behavior in different locations and helps identify areas that need further investigation, as well as assess whether there is any bias in the existing research.

Table 2 shows the number of articles published by country. From the table, the United Kingdom has the highest number of articles, with a total of 9 articles, followed by Switzerland with 6 articles. Other countries that also made significant contributions include Brazil and the United States, each with 4 articles. Meanwhile, countries such as the Netherlands, Poland, Pakistan, Austria, Turkey, Costa Rica, Cyprus, and Germany each have only 1 article.

**Table 2.** Number of Articles by Country.

Country	Total
Netherland	1
England	9
Brazil	4
Poland	1
Pakistan	1
USA	4
Switzerland	6
Austria	1
Turki	1
Costa Rica	1
Cyprus	1
Germany	1
Total	31

### Year of Publication

Figure 2 shows the distribution of articles by year of publication. In this graph, it is evident that the majority of articles were published in 2022, with 11 articles (gray color). Followed by 2023, which recorded 8 articles (orange color). The years 2020 and 2021 have 6 articles (blue color) and 5 articles (yellow color), respectively. Meanwhile, 2024 only has 1 article (light blue color). This graph provides a clear overview of the publication trends that occurred during the period from 2020 to 2024.

**Figure 2.**
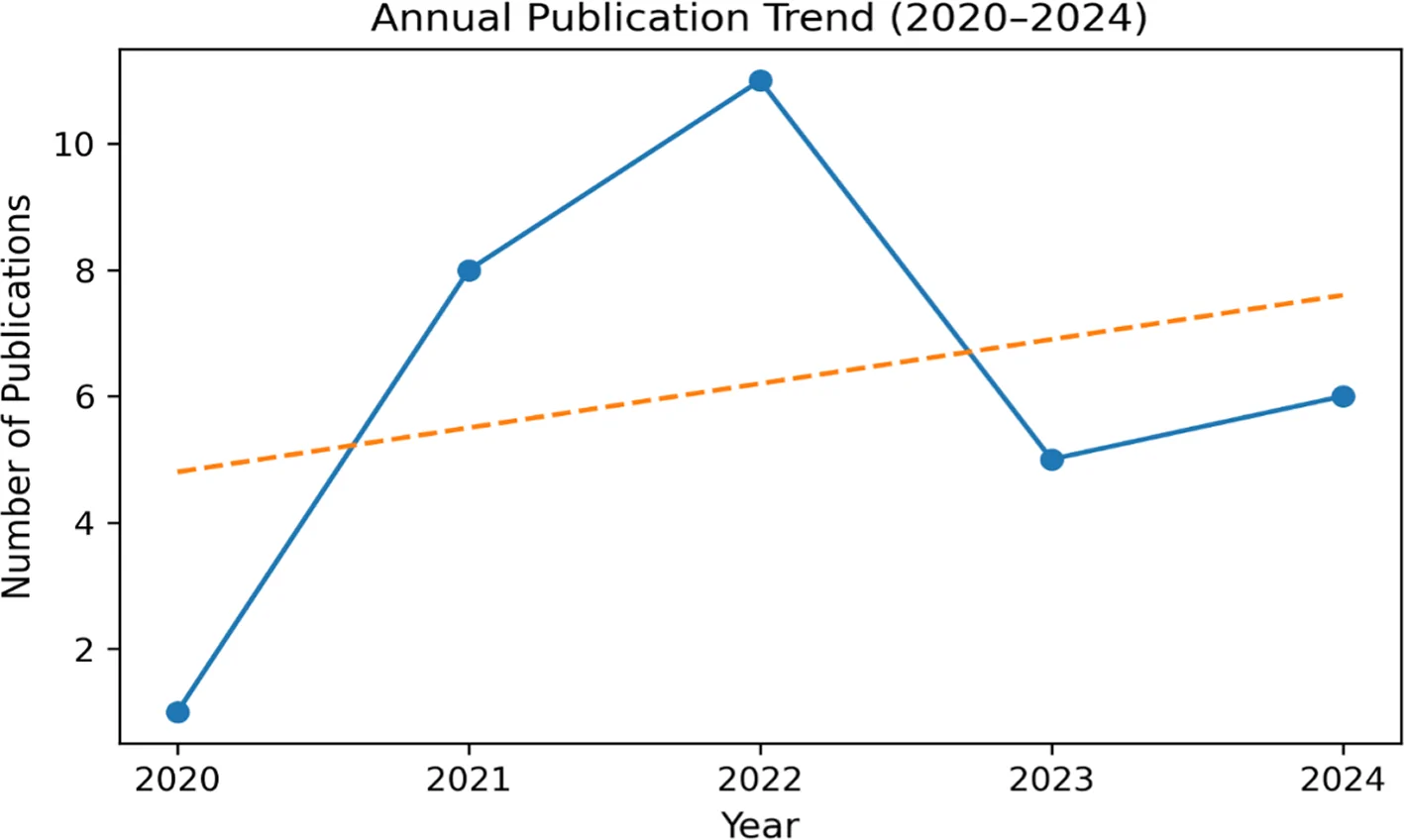
Keyword co-occurrence network of entrepreneurial behavior research. The visualization, generated using VOSviewer, shows the relationships among key terms, highlighting major research clusters such as entrepreneurship, student behavior, entrepreneurial intention, and contextual factors.

### Entrepreneurial Behaviour

The article search in the Scopus database for the period from 2020 to 2024 focus to the word “Entrepreneurial Behaviour” in the title. The goal of this analysis is to identify and analyze the terms frequently used in research related to student entrepreneurial behavior and entrepreneurship in general. This diagram aims to bridge the gap between general entrepreneurial behavior and the specific entrepreneurial behavior of students, which is the main focus of this article.

The analysis of the relationship between research titles was conducted using the VosViewer application, producing an output as shown in Figure 3. Student Entrepreneurial Behaviour (SEB) appears as one of the clusters related to entrepreneurial behaviour. Visually, Student Entrepreneurial Behaviour (SEB) is connected with several keywords such as student, entrepreneurial behaviour, and entrepreneurship education. This indicates that student entrepreneurial behaviour is a more specific theme within the broader context of entrepreneurial behaviour. In this diagram, Student Entrepreneurial Behaviour (SEB) is positioned in the center, surrounded by other keywords related to entrepreneurial intention, entrepreneurship education, and factors influencing student behaviour in the context of entrepreneurship. Its position tends to be in the middle or slightly toward the bottom right, close to keywords such as study, university student, and entrepreneurial education, suggesting that this topic is closely linked to the influence of education and university experience on students’ entrepreneurial intentions and actions.

**Figure 3.**
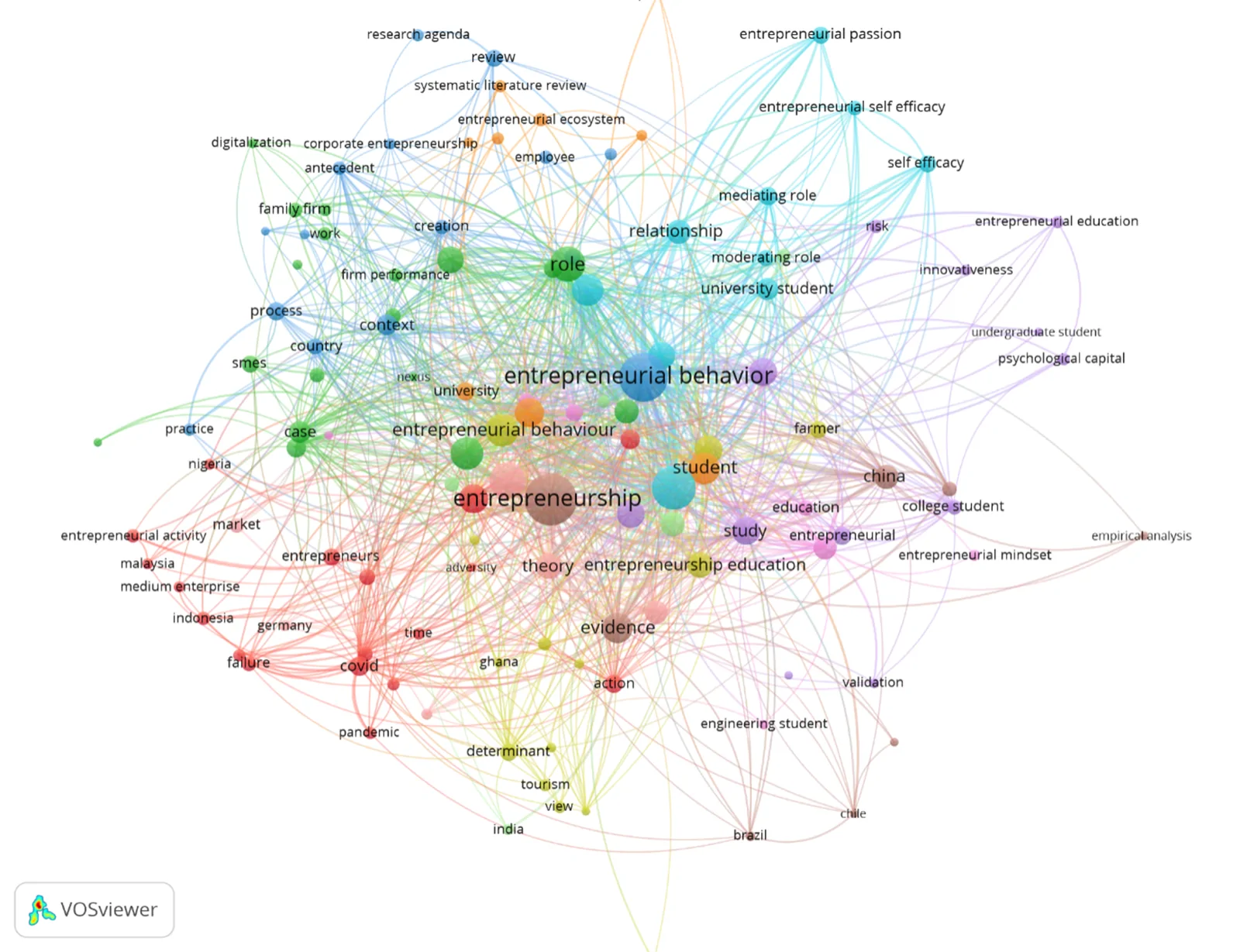
Thematic structure and relationships among key variables in student entrepreneurial behavior research. The network visualization illustrates the linkage between entrepreneurial behavior, entrepreneurial intention, student factors, and influencing variables, indicating the conceptual structure of the research field.

### Student Entrepreneurial Behaviour (SEB)

Focusing on the Student Entrepreneurial Behavior (SEB) keywords, a mapping of the relationship between keywords in articles related to Student Entrepreneurial Behavior (SEB) was also conducted. The same method and criteria used for the EB keywords were applied, resulting in a different mapping outcome. The results can be seen in Table 3. Figure 4 illustrates the keyword co-occurrence network for Student Entrepreneurial Behavior (SEB), highlighting the relationships among key research themes.

**Table 3.** Statistics on Keywords and Frequency of Appearance.

Cluster	Keywords and Frequency of Appearance	Theme
Cluster 1 (Red)	analysis, China, entrepreneurship education, evidence, social medium, student entrepreneurship, vocational college student	Study of the factors related to the students themselves that influence entrepreneurial intention
Cluster 2 (Green)	college student, education, entrepreneurial behavior, impact, theory	The influence of education and innovation on the development of students’ entrepreneurial intentions
Cluster 3 (Blue)	entrepreneurial intention, factor, influence, university	Applying the theory of planned behavior to study the factors influencing students’ social entrepreneurial intentions in higher education
Cluster 4 (Yellow)	Brazil, entrepreneurship, student	The impact of entrepreneurship education on the entrepreneurial mindset and startup activities of university students
Cluster 5 (Purple)	entrepreneurial behavior, scale validation, study	The measurement and validation of tools for assessing entrepreneurial behavior
Cluster 6 (Pink)	empirical analysis, students entrepreneurial behavior, university student	Empirical studies on students’ entrepreneurial behavior in university settings

**Figure 4.**
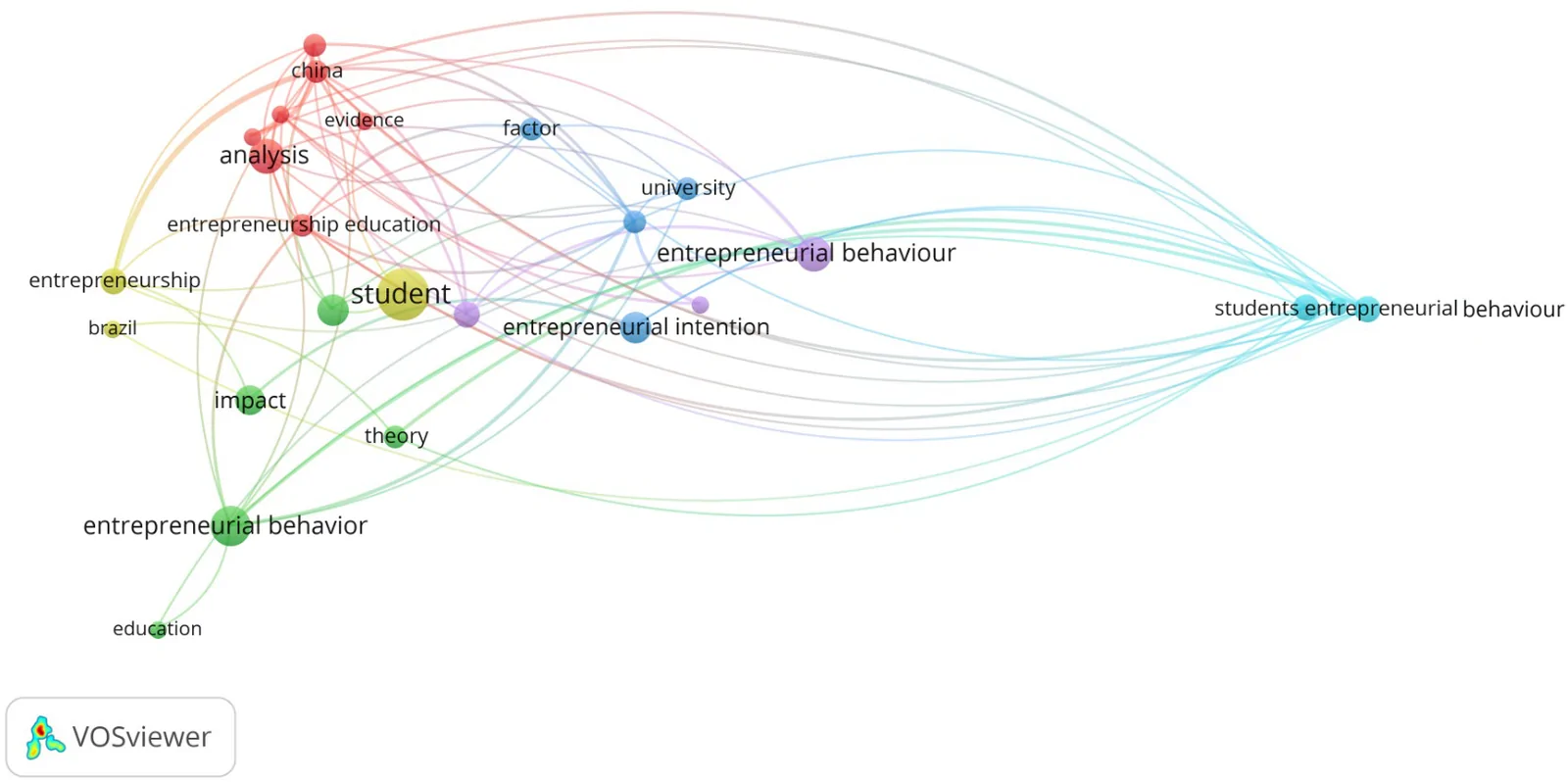
Keyword co-occurrence network of Student Entrepreneurial Behavior (SEB), showing thematic clusters and relationships among key research topics.

From this analysis, we can observe that research on Student Entrepreneurial Behaviour (SEB) focuses on various important aspects, ranging from entrepreneurship education, student behavior, entrepreneurial intention, to the social and cultural influences in specific countries. This diagram shows a strong relationship between education, social influences, and Student Entrepreneurial Behavior (SEB). It also highlights the importance of scale validation and empirical analysis in measuring the success and impact of entrepreneurship programs on students.

For a more in-depth analysis of Student Entrepreneurial Behavior (SEB), clusters that are considered closely related or adjacent were merged. In this case, the researcher combined Cluster 2 (Green) and Cluster 3 (Blue) into one cluster, as both focus on the influence of entrepreneurship education on students’ entrepreneurial intentions, as well as how the Theory of Planned Behavior is applied to analyze entrepreneurial intentions. Next, the Student Entrepreneurial Behavior (SEB) articles will be discussed according to the 5 clusters. These 5 clusters are:

1. Focus on Individual factors influencing SE intention
2. Focus on the influence of entrepreneurship education and entrepreneurial intention
3. Focus on social influences and external factors affecting Student Entrepreneurial Behavior (SEB)
4. Focus on validation methodology and scale in entrepreneurship research
5. Focus on empirical analysis and measurement of Student Entrepreneurial Behavior (SEB) in universities

## Discussions

The findings of this study should be interpreted within the context of its exploratory design. While the results offer meaningful insights into the emerging structure of Student Entrepreneurial Behavior (SEB) research, caution is warranted when generalizing beyond the analyzed dataset.

The discussion is structured around the five clusters identified in the previous analysis. Each cluster is examined through a systematic categorization of the included articles based on their primary focus, as summarized in Table 4.

**Table 4.** Article Adjustment According to Clusters.

Author	Cluster				
	1	2	3	4	5
( Cuong, 2023)	✔	✔			✔
( F. Wang & Sahid, 2024a)	✔			✔	✔
( Campos et al., 2021)		✔	✔		✔
( Fitouri, 2023)	✔	✔			✔
( Patel et al., 2024)	✔	✔	✔		
( Rocha et al., 2022)			✔		✔
( Echchabi et al., 2022)	✔			✔	
( Saupi et al., 2022)		✔			
( Sedegah et al., 2024)	✔		✔		
( Barreto Colichi et al., 2021)		✔			
( Ayob, 2021)	✔	✔			
( Bejinaru et al., 2023)		✔			
( Jiatong et al., 2022)		✔			
( Nam & Xiong, 2021)	✔				
( Oktavio et al., 2023)	✔				
( Zhang et al., 2022)			✔		
( Zhao, 2022)	✔	✔	✔		✔
( Bi & Collins, 2022)	✔	✔		✔	✔
( Majeed et al., 2021)	✔	✔		✔	✔
( Zhu & Zou, 2022)	✔	✔		✔	
( Almeida & Daniel, 2022)	✔	✔	✔		✔
( Soba et al., 2021)	✔	✔		✔	✔
( Yang, 2022)	✔	✔		✔	✔
( Cheng et al., 2022)	✔	✔		✔	✔
( Alakaleek et al., 2023)	✔	✔		✔	
( Liang et al., 2024)	✔	✔	✔		✔
( M. A. Fauzi et al., 2021)	✔	✔		✔	✔
( Ataei et al., 2021)	✔	✔		✔	✔
( Murad et al., 2024a)	✔	✔	✔		✔
( Aguirre-González et al., 2020)	✔	✔		✔	✔
( F. Wang & Sahid, 2024b)	✔	✔		✔	✔
Total	24	24	9	13	18

Cluster 1 and Cluster 2 contain the most articles, indicating that the topics of entrepreneurship education and student entrepreneurial behavior are the main focus in current research. Clusters 3, 4, and 5 are less frequently researched but still relevant in exploring social entrepreneurial intention, research methodology, and empirical analysis.

In Cluster 1, most research still focuses on the influence of self-efficacy, passion, and creativity on students’ entrepreneurial intentions ( Cuong, 2023; Fitouri, 2023). However, there is still a significant gap in examining how personal characteristics of students, such as risk propensity and intrinsic motivation, can influence their entrepreneurial decision-making in a broader context, especially in developing countries ( Ayob, 2021; Campos et al., 2021). Research on the differences in the influence of individual factors based on students’ cultural, social, and economic backgrounds is also limited ( Barreto Colichi et al., 2021; Jiatong et al., 2022). Future studies can be conducted by developing a more comprehensive model that integrates personal factors such as intrinsic motivation, risk propensity, and entrepreneurial mindset ( Alakaleek et al., 2023; Murad et al., 2024a). Other research could focus on cross-cultural comparisons to identify differences in the influence of individual factors on entrepreneurial intention in countries with different socio-economic conditions. Furthermore, research could explore the interaction between individual factors and entrepreneurship education support in encouraging students to take entrepreneurial steps.

In Cluster 2, although many studies has researched the influence of entrepreneurship education on students’ entrepreneurial intentions, there is still a gap in exploring the most effective teaching methods as well as external factors that either reinforce or hinder the impact of education. Most studies tend to overlook the social and cultural factors that may affect how much entrepreneurship education influences students, especially in developing countries ( Barreto Colichi et al., 2021; Campos et al., 2021). Furthermore, the lack of research examining the connection between entrepreneurship curricula and real-world changes in students’ entrepreneurial behavior is also a gap that needs to be addressed (A. Fauzi, 2014; Jiatong et al., 2022). Future research should focus on evaluating the effectiveness of various entrepreneurship teaching methods, such as practical approaches (e.g., real-world projects or business incubators) compared to traditional theoretical approaches ( Wang & Sahid, 2024b). Additionally, cross-country research is needed to examine the differences in the impact of entrepreneurship education within different social and cultural contexts ( Zhao, 2022). This research could explore the relationship between entrepreneurship curricula taught at universities and the application of entrepreneurial knowledge by students in the job market, as well as how external support, such as government policies or industry support, can strengthen students’ entrepreneurial intentions and behavior ( Alakaleek et al., 2023; Murad et al., 2024a).

In Cluster 3, although many studies discuss the influence of social and environmental factors on students’ social entrepreneurial intentions, there is still a significant gap in understanding the impact of local culture on entrepreneurial decisions, especially in countries with different economic and social contexts ( Ayob, 2021; Zhao, 2022). Existing research often focuses on external influences such as family or peer support, but tends to overlook the role of government policies and broader social structures in shaping social entrepreneurship among students ( Jiatong et al., 2022). Future research could explore more deeply how government policies and social environments in countries with different economic contexts influence students’ social entrepreneurial intentions ( Campos et al., 2021). Additionally, research could adopt a qualitative approach to better understand how cultural values and social norms in specific countries can either strengthen or limit social entrepreneurship ( Fitouri, 2023). Research could also examine the role of communities or social networks in helping students develop social entrepreneurial ideas and their implementation in local contexts ( Alakaleek et al., 2023; Zhao, 2022).

In Cluster 4, although many studies have discussed scale validation for measuring entrepreneurial behavior, there is still a lack of development in creating a more comprehensive and internationally applicable measurement scale. Many existing measurement tools are only used in specific contexts, such as in higher education or only in certain countries, so there is no global validation that can be applied across different cultures and educational systems ( Soba et al., 2021). Additionally, there is a gap in measurement that does not solely rely on self-reporting from students, which can lead to bias in the measurement results ( Ataei et al., 2021). Future research needs to develop an entrepreneurship measurement scale that can be applied across various cultural contexts and educational systems. This scale should validate broader dimensions of entrepreneurship, encompassing not only entrepreneurial behavior but also social innovation and resilience. Research should also focus on alternative methodologies to reduce self-report bias in measurements, such as using observational methods or outcome-based measurements in students’ entrepreneurial practices (M. A. Fauzi et al., 2021; Jiatong et al., 2022; Soba et al., 2021).

In Cluster 5, although many studies examine Student Entrepreneurial Behavior (SEB) in universities, there is still a lack of longitudinal research that tracks changes in Students Entrepreneurial Behavior (SEB) over the long term. Many studies are cross-sectional, which limits the understanding of how entrepreneurial intentions develop and are translated into entrepreneurial actions over time ( Cuong, 2023). Furthermore, research still lacks a focus on external factors outside of universities that influence the success of student entrepreneurship, such as access to financing, mentoring, or professional networks ( Fitouri, 2023). Future research could adopt a longitudinal approach to study how students’ entrepreneurial behavior evolves from intention to actual action after they graduate and enter the workforce ( Zhao, 2022). Additionally, more in-depth research is needed to explore the influence of external support, such as access to financing, business incubators, and professional networks, on the success of student entrepreneurship beyond university. The research would provide a more comprehensive picture of the entrepreneurial ecosystem that can support students in realizing their business ideas after graduation ( Jiatong et al., 2022; Soba et al., 2021).

## Conclusion

This study provides an exploratory bibliometric overview of Student Entrepreneurial Behavior (SEB) research within higher education. The findings reveal that, despite growing scholarly interest, the field remains conceptually fragmented and methodologically limited, with a strong emphasis on entrepreneurial intention rather than observable behavior.

The study contributes by positioning Student Entrepreneurial Behavior (SEB) as a measurable educational outcome and by identifying key thematic areas and research gaps, particularly in relation to longitudinal analysis, cross-cultural validation, and behavioral measurement.

Future research should prioritize more rigorous methodological designs and integrative frameworks that capture the dynamic transition from intention to behavior. Strengthening this line of inquiry is essential for advancing entrepreneurship education and enhancing its practical impact on student outcomes.

### Limitations

This study should be interpreted in light of several limitations. First, the bibliometric dataset was restricted to publications indexed in the Scopus database and a relatively short time span (2020–2024), which reflects the emerging nature of Student Entrepreneurial Behavior (SEB) research but may limit the comprehensiveness of long-term trend analysis.

Second, the relatively small dataset (31 publications) indicates the specialized scope of the field and may constrain the generalizability of the findings.

Third, the use of title-based search criteria, while ensuring conceptual precision, may have excluded relevant studies that address Student Entrepreneurial Behavior (SEB) using alternative terminology.

Finally, the analysis relies primarily on descriptive bibliometric techniques and does not incorporate advanced citation-based methods, such as co-citation or bibliographic coupling, which could provide deeper insights into the intellectual structure of the field.

Accordingly, the findings of this study should be interpreted as exploratory and indicative rather than definitive, providing a foundation for more comprehensive and methodologically robust future research.

## Data Availability

The bibliometric dataset used in this study was obtained from the Scopus database. The dataset and analysis files supporting the findings of this study are openly available in the Zenodo repository: https://doi.org/10.5281/zenodo.19057716 ( Nasir et al., 2026). The dataset is shared under a Creative Commons Attribution 4.0 International (CC BY 4.0) license.
