# Supplementary material for: Student Entrepreneur Behavior (SEB) : A Bibliometric Analysis
Source: F1000Res. 2026 Aug 11;15:794. Originally published 2026 May 25. [Version 2] doi: 10.12688/f1000research.175425.2 (PMC13691362; doi:10.12688/f1000research.175425.2)
Supplement: Peer review history

# Reviewer report: Reviewer response for version 2

**DOI:** 10.5256/f1000research.207547.r528222

**Published:** 2026-10-22

**Authors:** Amjed Hameed Majeed

**Affiliations:** 1 Al-MustaqbalUniversity, Babylon, Iraq

1. General Assessment

 The study addresses a relevant and increasingly important topic in entrepreneurship education, particularly the distinction between entrepreneurial intention and actual entrepreneurial behavior among university students. This distinction represents a meaningful research problem, as much of the existing literature focuses on students' entrepreneurial intentions rather than their observable entrepreneurial actions.

 The proposed bibliometric analysis offers a potentially valuable contribution by mapping publication trends, thematic clusters, and research gaps in Student Entrepreneurial Behavior (SEB) research.

 The abstract is logically organized, academically appropriate, and generally clear. However, several methodological and conceptual issues require further clarification to strengthen the study's scientific rigor, transparency, reproducibility, and contribution to the literature.

 In particular, the limited dataset of 31 publications, the search and screening procedures, the operational definition of SEB, and the interpretation of bibliometric clusters require closer attention.

 2. Evaluation of the Abstract

 Evaluation CriterionAssessment

 Research relevanceStrong

 Clarity of research objectivesGood

 Originality and potential contributionPromising

 Methodological transparencyNeeds improvement

 Bibliometric analytical rigorNeeds clarification

 Interpretation of findingsModerately supported

 Recognition of limitationsGood

 Overall scientific contributionPotentially valuable

 3. Major Comments

 Comment 1: Conceptual Definition of Student Entrepreneurial Behavior

 The manuscript emphasizes the distinction between entrepreneurial intention and entrepreneurial behavior. Nevertheless, the abstract does not explain how Student Entrepreneurial Behavior is conceptually defined or operationally distinguished from entrepreneurial intention.

 This distinction is particularly important because studies addressing entrepreneurial intentions, entrepreneurial competencies, entrepreneurial engagement, and actual venture creation may be classified differently.

 Recommendation: The authors should provide a clear conceptual definition of SEB and specify the behavioral indicators used to determine whether publications are relevant to the analysis. Examples could include entrepreneurial opportunity recognition translated into action, venture initiation, entrepreneurial experimentation, and business creation activities.

 Comment 2: Limited Bibliometric Dataset

 The analysis includes only 31 Scopus-indexed publications between 2020 and 2024.

 Although a limited dataset does not automatically invalidate a bibliometric study, it raises questions regarding the comprehensiveness of the literature search and the stability of the identified thematic clusters.

 In addition, the abstract does not explain why the study period was restricted to five years.

 Recommendation: The authors should justify the selected publication period and sample size, clarify whether the dataset represents the full population of relevant publications identified using the search criteria, and explain how the small sample affects the interpretation of bibliometric findings.

 The exploratory nature of the study should be maintained consistently throughout the manuscript.

 Comment 3: Transparency and Reproducibility of the Search Strategy

 The abstract states that bibliographic data were retrieved using Publish or Perish and analyzed using VOSviewer.

 However, the search strategy is insufficiently described. Important details are missing, including the exact search terms, Boolean operators, date of data retrieval, inclusion and exclusion criteria, document types, and screening process.

 Recommendation: The authors should report a reproducible search strategy, including the database fields searched and the method used to verify that the retrieved records were indexed in Scopus.

 It would also be beneficial to provide a publication selection flow diagram and explain whether duplicate records and irrelevant publications were removed.

 Comment 4: Bibliometric Analytical Procedures

 The abstract identifies performance analysis and keyword co-occurrence mapping as the primary analytical approaches. However, the analytical settings and indicators are not specified.

 For a dataset of only 31 publications, thematic patterns may be particularly sensitive to keyword selection, normalization, and network thresholds.

 Recommendation: The authors should clarify the bibliometric indicators employed, the minimum keyword occurrence threshold, the counting method, keyword harmonization procedures, and the normalization approach used in VOSviewer.

 The authors should also explain how thematic clusters were identified and labeled.

 Comment 5: Interpretation of Research Findings

 The results indicate geographic concentration, fluctuating publication trends, and a predominance of cross-sectional studies.

 These observations are potentially important, but the abstract does not provide quantitative evidence to support them.

 For example, the reader cannot determine the extent of geographic concentration or the proportion of cross-sectional research designs.

 Furthermore, the predominance of cross-sectional studies is a methodological finding that generally requires examination of the underlying publications rather than bibliometric mapping alone.

 Recommendation: The authors should provide concise descriptive indicators, such as annual publication counts, leading countries, and the proportion of studies using cross-sectional designs.

 They should also distinguish findings derived from bibliometric analysis from those obtained through manual examination or content analysis.

 Comment 6: The Intention–Behavior Gap

 The continuing intention–behavior gap is presented as a key finding and conclusion.

 However, the abstract does not establish whether this gap was directly measured or identified through the thematic and conceptual characteristics of the reviewed literature.

 Bibliometric analysis can identify patterns of scholarly attention, but it does not independently demonstrate the magnitude of the intention–behavior gap.

 Recommendation: The authors should clarify whether the intention–behavior gap is an empirically established finding of the analyzed publications or an identified gap in research coverage.

 If it is the latter, the conclusion should use more cautious terminology.

 Comment 7: Theoretical and Educational Contributions

 The conclusion suggests that the findings may support evidence-informed curriculum development in entrepreneurship education.

 This implication is relevant but currently underdeveloped.

 The abstract does not specify how the bibliometric findings could inform entrepreneurship curricula or educational interventions.

 Recommendation: The authors should identify more explicitly how future entrepreneurship education research could move beyond intention-based indicators toward behavioral outcomes, such as entrepreneurial activities, experimentation, and venture-related engagement.

 Such clarification would strengthen the practical significance of the study.

 4. Minor Comments

 1. Terminological consistency: Use the term Student Entrepreneurial Behavior (SEB) consistently and clarify whether the study focuses exclusively on university students or broader higher education populations.

 2. Methodological precision: The statement that data were retrieved using Publish or Perish should specify its role as a retrieval interface and the procedure used to establish the Scopus indexing status of records.

 3. Results presentation: The phrase “several major research areas” could be strengthened by reporting the number of thematic clusters identified, if established by the analysis.

 4. Originality: The abstract should state more explicitly what distinguishes the study from previous bibliometric reviews of entrepreneurship education and entrepreneurial intentions.

 5. Conclusion: The recommendation for longitudinal, mixed-method, and cross-cultural studies is appropriate, but should be linked more explicitly to observed limitations within the analyzed dataset.

 6. Academic style: The abstract is well written overall. Some repeated use of the full term Student Entrepreneurial Behavior (SEB) could be reduced after the abbreviation is introduced.

 5. Strengths of the Study

 The manuscript demonstrates several positive features.

 First, it focuses on entrepreneurial behavior rather than entrepreneurial intention alone, which provides a meaningful direction for entrepreneurship education research.

 Second, the integration of performance analysis and keyword co-occurrence mapping may offer a useful overview of the intellectual structure of this emerging research area.

 Third, the identification of individual, educational, and social dimensions suggests the potential for developing a more integrated understanding of entrepreneurial behavior among students.

 Finally, the authors appropriately acknowledge the limited publication sample and characterize their findings as exploratory. This demonstrates awareness of the study's methodological limitations.

 6. Recommendations for Improvement

 The following revisions should receive priority:

 PriorityRequired Revision

 HighProvide a precise conceptual and operational definition of SEB

 HighReport the complete literature search and screening methodology

 HighJustify the dataset of 31 publications and the 2020–2024 study period

 HighExplain bibliometric network settings and thematic cluster identification

 HighDistinguish bibliometric results from methodological content analysis

 MediumSupport reported trends with descriptive quantitative evidence

 MediumClarify the interpretation of the intention–behavior gap

 MediumStrengthen theoretical originality and educational implications

 7. Final Reviewer Comments to the Authors

**Reviewer Comments to the Authors**

 The manuscript addresses a timely and relevant topic in entrepreneurship education by examining the development of Student Entrepreneurial Behavior (SEB) research. Its emphasis on observable entrepreneurial behavior, rather than entrepreneurial intention alone, is particularly valuable and has the potential to contribute meaningfully to this field.

 The structured abstract is generally clear, coherent, and appropriately organized. The exploratory bibliometric approach is suitable for providing an initial overview of publication trends and thematic patterns, particularly if the authors seek to identify emerging research directions.

 However, several aspects require substantial clarification before the study's scientific contribution can be adequately assessed.

 First, the conceptual boundaries of Student Entrepreneurial Behavior should be explicitly defined to distinguish behavioral outcomes from intentions, attitudes, and entrepreneurial competencies.

 Second, greater methodological transparency is required regarding the selection of the 31 Scopus-indexed publications, the justification for the 2020–2024 study period, and the bibliometric procedures implemented using Publish or Perish and VOSviewer.

 Third, the authors should provide stronger quantitative support for the reported findings, particularly those concerning geographic concentration, publication fluctuations, and the prevalence of cross-sectional research designs.

 Fourth, the interpretation of the intention–behavior gap requires clarification, since bibliometric patterns alone cannot establish the empirical magnitude of this relationship.

 Finally, the manuscript would benefit from a more explicit explanation of its theoretical contribution and its implications for entrepreneurship curriculum development and future behavioral research.

 Overall, the study offers a promising exploratory contribution. Nevertheless, improvements in conceptual clarity, methodological reproducibility, analytical transparency, and interpretation of findings are necessary to strengthen its academic rigor and potential publication value.

**Recommendation: Major Revision.**

 8. Final Recommendation

 Major Revision

 The study presents a relevant research question and a potentially useful exploratory analysis. However, the abstract does not yet provide sufficient methodological detail or quantitative evidence to support all reported findings confidently.

 The recommendation is therefore to undertake major revisions, primarily focused on conceptual precision, search reproducibility, bibliometric analytical procedures, and the distinction between observed results and broader research implications.

Is the work clearly and accurately presented and does it cite the current literature?

Yes

If applicable, is the statistical analysis and its interpretation appropriate?

Yes

Are all the source data underlying the results available to ensure full reproducibility?

Yes

Is the study design appropriate and is the work technically sound?

Yes

Are the conclusions drawn adequately supported by the results?

Yes

Are sufficient details of methods and analysis provided to allow replication by others?

Yes

Reviewer Expertise:

business administration

I confirm that I have read this submission and believe that I have an appropriate level of expertise to confirm that it is of an acceptable scientific standard, however I have significant reservations, as outlined above.

# Reviewer report: Reviewer response for version 1

**DOI:** 10.5256/f1000research.193407.r498763

**Published:** 2026-07-10

**Authors:** Asmaa Abdul Wahid Malik

**Affiliations:** 1 AI-Furat Al-Awsat Technical University, Kufa, Iraq

I thank the editorial board for giving me the opportunity to review this article. After reviewing it, I would like to summarize my evaluation, highlighting its strengths and offering some observations to enhance its quality:

 Strengths:

 The work was presented clearly and accurately, citing recent scientific references. The research was well-structured, beginning with the methodology, followed by descriptive statistics, citations, and the use of maps, figures, and appropriate tools for biometric analysis. The study's objective was also clear in terms of the relationship between entrepreneurial learning, entrepreneurial mindset, and entrepreneurial behavior.

 Furthermore, the study design is technically sound in terms of its organization and the use of appropriate tools and figures.

 Despite these strengths, there are some observations, namely:

 First, the methodology details were only partially provided. The researcher used a single database and limited the sample to a small number of studies from 2020-2024 (31 studies), in addition to only two replications.

 Second, the statistical analysis was appropriate, and the researcher used descriptive analysis, which is the most suitable approach for biometric studies in terms of using appropriate tools, citations, and keyword frequency. The interpretation of the results was adequate but lacked sufficient depth. The researcher did not provide a satisfactory explanation for the higher results in some years or countries compared to others, nor for the lower results and variations in other countries. For example, could this be due to the development of entrepreneurship in some places, the availability of necessary resources, or the greater ability and desire for development among students in different locations?

 Thirdly, regarding the availability of source data and the possibility of partial replication, the researcher did specify the database used, the time period, the number of studies, the testing tools, figures, and maps. However, the researcher did not include the original data file before processing, nor did they explain the data processing method used in the analysis and the resulting findings.

 Fourthly, the conclusions are generally consistent with the results, but some require further elaboration, such as linking certain conclusions to specific numerical results or percentages. This is my sincere thanks and appreciation.

Is the work clearly and accurately presented and does it cite the current literature?

Yes

If applicable, is the statistical analysis and its interpretation appropriate?

Partly

Are all the source data underlying the results available to ensure full reproducibility?

Partly

Is the study design appropriate and is the work technically sound?

Yes

Are the conclusions drawn adequately supported by the results?

Yes

Are sufficient details of methods and analysis provided to allow replication by others?

Partly

Reviewer Expertise:

Theory and Organizational Behavior

I confirm that I have read this submission and believe that I have an appropriate level of expertise to confirm that it is of an acceptable scientific standard.

# Response

**Published:** 2026-07-30

**Authors:** Munawir Nasir Hamzah

**Affiliations:** Doctor Research of Management, BINUS University, West Jakarta, Jakarta, Indonesia

Thank you for your detailed and constructive review. We have addressed all major and minor comments point-by-point in the attached Response to Reviewers document (uploaded with this new version) and revised the manuscript accordingly, including corrections to publication-year and cluster-count inconsistencies, expanded methodological detail, a softened novelty claim, and an updated Zenodo repository with the requested PRISMA flow diagram and raw data files.

# Reviewer report: Reviewer response for version 1

**DOI:** 10.5256/f1000research.193407.r490657

**Published:** 2026-06-19

**Authors:** Mathew Thomas Gil

**Affiliations:** 1 Manipal Academy of Higher Education, Manipal, Karnataka, India

My comments below are intended to help you bring the article to a state where its results can be verified and its method defended; they are grouped into major, minor, and editorial points.

 Strengths

- A timely, clearly motivated focus, with a helpful conceptual distinction between entrepreneurial intention and enacted behaviour as a learning outcome.
- A transparently reported search string and screening sequence, and a candid limitations section that acknowledges the small corpus and exploratory scope.
- A data-availability statement pointing to a Zenodo deposit  consistent with F1000’s open-data expectation (see major point 6 on confirming it).

Major comments

1. Reconcile the contradictory year-of-publication figures. The Methods (scope-limitation stage) report the corpus as 1 article in 2020, 8 in 2021, 11 in 2022, 5 in 2023, and 6 in 2024. The Results (Year of Publication) instead report 6 in 2020, 5 in 2021, 11 in 2022, 8 in 2023, and 1 in 2024. Both sum to 31, but the distributions are mutually inconsistent (e.g., 2020 is 1 vs 6; 2024 is 6 vs 1). A reader cannot tell which is correct. Please correct this and re-check every count in the descriptive section against the dataset.
2. Resolve the cluster count and the merging step. The text states that clusters were merged into five clusters and lists five, but Table 3 presents six clusters (Red through Pink), and Table 4 uses five cluster columns. Please present a single, internally consistent cluster solution, state clearly which clusters were merged and why, and ensure the narrative, Table 3, and Table 4 all agree.
3. Fix the figure numbering and ensure the visualisations are present and legible. Figure 2 is described in one place as the distribution of articles by year and in another as a keyword co-occurrence network; Figures 3 and 4 are both described as co-occurrence networks. Each figure must be uniquely numbered, captioned, and actually rendered. Because the VOSviewer maps are central to a bibliometric paper, please ensure they are legible and that the parameters (counting method, occurrence threshold, normalisation, resolution) are reported so the maps can be reproduced.
4. Justify the bibliometric approach given the very small corpus, and reframe the claims. Thirty-one title-screened documents is a small basis for bibliometric mapping, and keyword co-occurrence built from titles only (minimum occurrence of two) yields sparse, sensitive maps. The broad “entrepreneurial behaviour” search underlying Figures 2–3 was also truncated at 200 records per year by the software, so it is not exhaustive, and the “citation-based relevance and H-index” filtering is not specified reproducibly. Please justify the design, consider drawing on author keywords / abstract terms rather than titles alone, and reframe outputs as illustrative rather than as a definitive “intellectual structure” or “thematic evolution.” For a corpus this size, a scoping or systematic review with qualitative synthesis may be more defensible than VOSviewer mapping; at minimum, temper the language accordingly.
5. Temper the novelty claim and engage prior bibliometric work. The article states that no comprehensive bibliometric analysis has framed student entrepreneurial behaviour. However, closely related bibliometric studies exist — for example, large analyses of college students’ entrepreneurial intention (several hundred articles) and recent bibliometric work explicitly on entrepreneurial intention and behaviour. Please cite and position your contribution against this body of work, and reconcile your stated exclusion of “intention-only” studies with the corpus, which includes several titles explicitly about entrepreneurial intention.
6. Confirm and complete the open dataset. F1000Research requires accessible underlying data. The statement cites a Zenodo DOI, but it is mis-formatted (it contains a space), and I was unable to confirm that it resolves. Please verify that the record is public and that it contains everything needed to reproduce the study the RIS export of the 31 records, the VOSviewer map/network files, and the screening log and correct the DOI so it resolves directly.

Minor comments

1. There is an erroneous reference: “Fauzi A (2014)” on Base Transceiver Station (BTS) placement and telecommunications towers is cited in the discussion of entrepreneurship curricula. This is clearly the wrong source — please remove or replace it, and verify the full reference list against the in-text citations.
2. Clarify what “country” denotes in Table 2. “Country of the publisher” conflates the publisher’s location with the research’s origin (e.g., Switzerland ”6” likely reflects publisher headquarters such as MDPI/Frontiers rather than where the studies were conducted). Use a consistent, defensible attribution (author affiliation or corresponding-author country) and label it accordingly.
3. The article refers to “the SLR articles,” but no systematic-review protocol (e.g., a PRISMA flow) is reported. Please use consistent terminology either present this as a bibliometric study or, if claiming systematic screening, include the corresponding flow diagram.
4. Tie the stated research gaps (longitudinal designs, cross-cultural validation, behavioural measurement) more explicitly to the bibliometric results; at present they read as drawn from the narrative literature rather than derived from the maps.
5. Standardise country names (“Netherland” → Netherlands; “Turki” → Turkey; “England” vs “United Kingdom”) and confirm Table 2 against the corpus.

Is the work clearly and accurately presented and does it cite the current literature?

Partly

If applicable, is the statistical analysis and its interpretation appropriate?

Not applicable

Are all the source data underlying the results available to ensure full reproducibility?

Partly

Is the study design appropriate and is the work technically sound?

Partly

Are the conclusions drawn adequately supported by the results?

Partly

Are sufficient details of methods and analysis provided to allow replication by others?

Partly

Reviewer Expertise:

Entrepreneurship, sustainability, marketing

I confirm that I have read this submission and believe that I have an appropriate level of expertise to confirm that it is of an acceptable scientific standard, however I have significant reservations, as outlined above.

# Response

**Published:** 2026-07-30

**Authors:** Munawir Nasir Hamzah

**Affiliations:** Doctor Research of Management, BINUS University, West Jakarta, Jakarta, Indonesia

*Thank you for your detailed and constructive review. We have addressed all major and minor comments point-by-point in the attached Response to Reviewers document (uploaded with this new version) and revised the manuscript accordingly, including corrections to publication-year and cluster-count inconsistencies, expanded methodological detail, a softened novelty claim, and an updated Zenodo repository with the requested PRISMA flow diagram and raw data files.*
